# Distinct Fecal and Plasma Metabolites in Children with Autism Spectrum Disorders and Their Modulation after Microbiota Transfer Therapy

**DOI:** 10.1128/mSphere.00314-20

**Published:** 2020-10-21

**Authors:** Dae-Wook Kang, James B. Adams, Troy Vargason, Marina Santiago, Juergen Hahn, Rosa Krajmalnik-Brown

**Affiliations:** a Biodesign Swette Center for Environmental Biotechnology, Arizona State University, Tempe, Arizona, USA; b School for Engineering of Matter, Transport and Energy, Arizona State University, Tempe, Arizona, USA; c Department of Biomedical Engineering, Rensselaer Polytechnic Institute, Troy, New York, USA; d Center for Biotechnology and Interdisciplinary Studies, Rensselaer Polytechnic Institute, Troy, New York, USA; e Finch Therapeutics, Somerville, Massachusetts, USA; f Department of Chemical and Biological Engineering, Rensselaer Polytechnic Institute, Troy, New York, USA; g School of Sustainable Engineering and the Built Environment, Arizona State University, Tempe, Arizona, USA; h Biodesign Center for Health Through Microbiomes, Arizona State University, Tempe, Arizona, USA; University of California, Davis

**Keywords:** autism spectrum disorder (ASD), clinical trial, fecal microbiota transplant (FMT), gut bacteria, metabolites, microbiome, microbiota transplant therapy (MTT)

## Abstract

Accumulating evidence has strengthened a link between dysbiotic gut microbiota and autism. Fecal microbiota transplant (FMT) is a promising therapy to repair dysbiotic gut microbiota. We previously performed intensive FMT called microbiota transfer therapy (MTT) for children with autism spectrum disorders (ASD) and observed a substantial improvement of gastrointestinal and behavioral symptoms. We also reported modulation of the gut microbiome toward a healthy one. In this study, we report comprehensive metabolite profiles from plasma and fecal samples of the children who participated in the MTT trial. With 619 plasma metabolites detected, we found that the autism group had distinctive metabolic profiles at baseline. Eight metabolites (nicotinamide riboside, IMP, iminodiacetate, methylsuccinate, galactonate, valylglycine, sarcosine, and leucylglycine) were significantly lower in the ASD group at baseline, while caprylate and heptanoate were significantly higher in the ASD group. MTT drove global shifts in plasma profiles across various metabolic features, including nicotinate/nicotinamide and purine metabolism. In contrast, for 669 fecal metabolites detected, when correcting for multiple hypotheses, no metabolite was significantly different at baseline. Although not statistically significant, *p*-cresol sulfate was relatively higher in the ASD group at baseline, and after MTT, the levels decreased and were similar to levels in typically developing (TD) controls. *p*-Cresol sulfate levels were inversely correlated with *Desulfovibrio*, suggesting a potential role of *Desulfovibrio* on *p*-cresol sulfate modulation. Further studies of metabolites in a larger ASD cohort, before and after MTT, are warranted, as well as clinical trials of other therapies to address the metabolic changes which MTT was not able to correct.

**IMPORTANCE** Despite the prevalence of autism and its extensive impact on our society, no U.S. Food and Drug Administration-approved treatment is available for this complex neurobiological disorder. Based on mounting evidences that support a link between autism and the gut microbiome, we previously performed a pioneering open-label clinical trial using intensive fecal microbiota transplant. The therapy significantly improved gastrointestinal and behavioral symptoms. Comprehensive metabolomic measurements in this study showed that children with autism spectrum disorder (ASD) had different levels of many plasma metabolites at baseline compared to those in typically developing children. Microbiota transfer therapy (MTT) had a systemic effect, resulting in substantial changes in plasma metabolites, driving a number of metabolites to be more similar to those from typically developing children. Our results provide evidence that changes in metabolites are one mechanism of the gut-brain connection mediated by the gut microbiota and offer plausible clinical evidence for a promising autism treatment and biomarkers.

## Abstract (precis)

Despite the prevalence of autism and its extensive impact on our society, no U.S. Food and Drug Administration-approved treatment is available for this complex neurobiological disorder. Based on mounting evidences that support a link between autism and the gut microbiome, we previously performed a pioneering open-label clinical trial using intensive fecal microbiota transplant. The therapy significantly improved gastrointestinal and behavioral symptoms. Comprehensive metabolomic measurements in this study showed that children with autism spectrum disorder (ASD) had different levels of many plasma metabolites at baseline compared to those in typically developing children. Microbiota transfer therapy (MTT) had a systemic effect, resulting in substantial changes in plasma metabolites, driving a number of metabolites to be more similar to those from typically developing children. Our results provide evidence that changes in metabolites are one mechanism of the gut-brain connection mediated by the gut microbiota and offer plausible clinical evidence for a promising autism treatment and biomarkers.

## INTRODUCTION

In the last decade, the world has seen a substantial increase in the diagnosis of autism spectrum disorder (ASD) (1, 2). “Autism” is a label given to children with certain significant impairments in social communication and behavior (3). However, unlike disorders such as diabetes which have a known biochemical mechanism and biomarker (abnormal blood sugar), there is no general consensus of the biochemical basis of the condition(s) known as ASD. One potentially important etiology could be related to the gut-brain axis and gut microbiome (4). Notably, many children with ASD have chronic gastrointestinal (GI) problems (constipation and/or diarrhea) (5, 6). Those GI problems have been associated with a higher severity of ASD (7) and triggered questions on a possible link between the gut microbiome and ASD. Many studies revealed that individuals with ASD have different gut microbiome compositions than typically developing (TD) individuals (8–13), and several studies found higher levels of certain pathogenic clostridia species or lower levels of potentially beneficial microbes in children with ASD than in TD children (14–16).

Different studies of the microbiome in children with ASD have sometimes yielded conflicting results (17). This is partly due to small sample sizes, different measurement methods, and sometimes inadequate statistical correction for multiple-hypothesis testing. However, part of the reason might be also due to microbes’ diverse repertoires of metabolic functions. Any particular microbe may be either beneficial or detrimental, depending on its environment or other community members. For example, *Desulfovibrio* produces lipopolysaccharides (LPS) and hydrogen sulfide, metabolites that could be toxic to the integrity of gut epithelial cells of individuals with ASD (18), but it also consumes hydrogen gas and contributes to energetically efficient fermentation to generate ATP and produce short-chain fatty acids (19, 20). Furthermore, microbes respond to changes in their environment and interact with each other by either competing or collaborating, and in many cases, microbes share the same metabolism; this is called functional redundancy. Therefore, to better understand the difference in ASD microbiome function, it is useful to investigate the combined functional repertoires of the whole gut microbiota using “metabolomics” rather than rely solely on the presence or abundances of specific microbial species.

A number of studies have reported that metabolite profiles in fecal, urine, and blood samples from individuals with ASD are different than the metabolite profiles of typically developing children without known disorders (8, 21, 22), children without a family history of autism and unaffected typically developing siblings (21, 23, 24), and healthy adults (25). The level of an excitatory neurotransmitter, glutamate, has been reported to be higher in feces and blood from individuals with autism (8, 21). An increased ratio of glutamate to gamma-aminobutyric acid (GABA) is known to be a signature of neuroinflammation linked to sensory processing, and the ratio between these two major neurotransmitters was reported to be different in blood samples from children with ASD compared to those from TD children (23). Glutathione is an essential metabolite for detoxification, and its redox ratio between reduced and oxidized glutathione has also been reported to be disrupted in individuals with ASD compared with that in controls (22). Several studies reported significantly higher levels of *p*-cresol, a tyrosine derivative, in blood, urine, and feces from children with ASD (8, 24, 25). Experiments with a maternal immune activation ASD mouse model revealed that 4-ethylphenyl sulfate (4-EPS) and indolepyruvate, two tyrosine/tryptophan derivatives, were significantly higher in mice that had ASD symptoms, and the concentration of 4-EPS decreased with Bacteroides fragilis administration (26). In recent ASD mouse model experiments, taurine and 5-aminovaleric acid were lower in colon contents from ASD mice, and when these two GABA receptor agonists were administered to ASD mice, their ASD-like behaviors improved (27).

**FIG 1:**
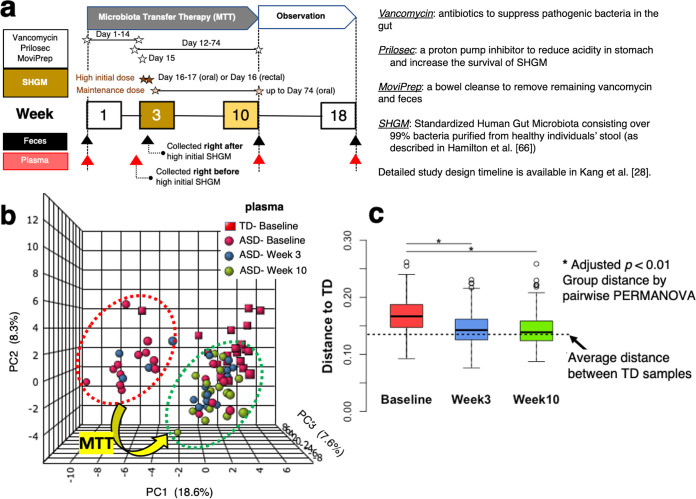

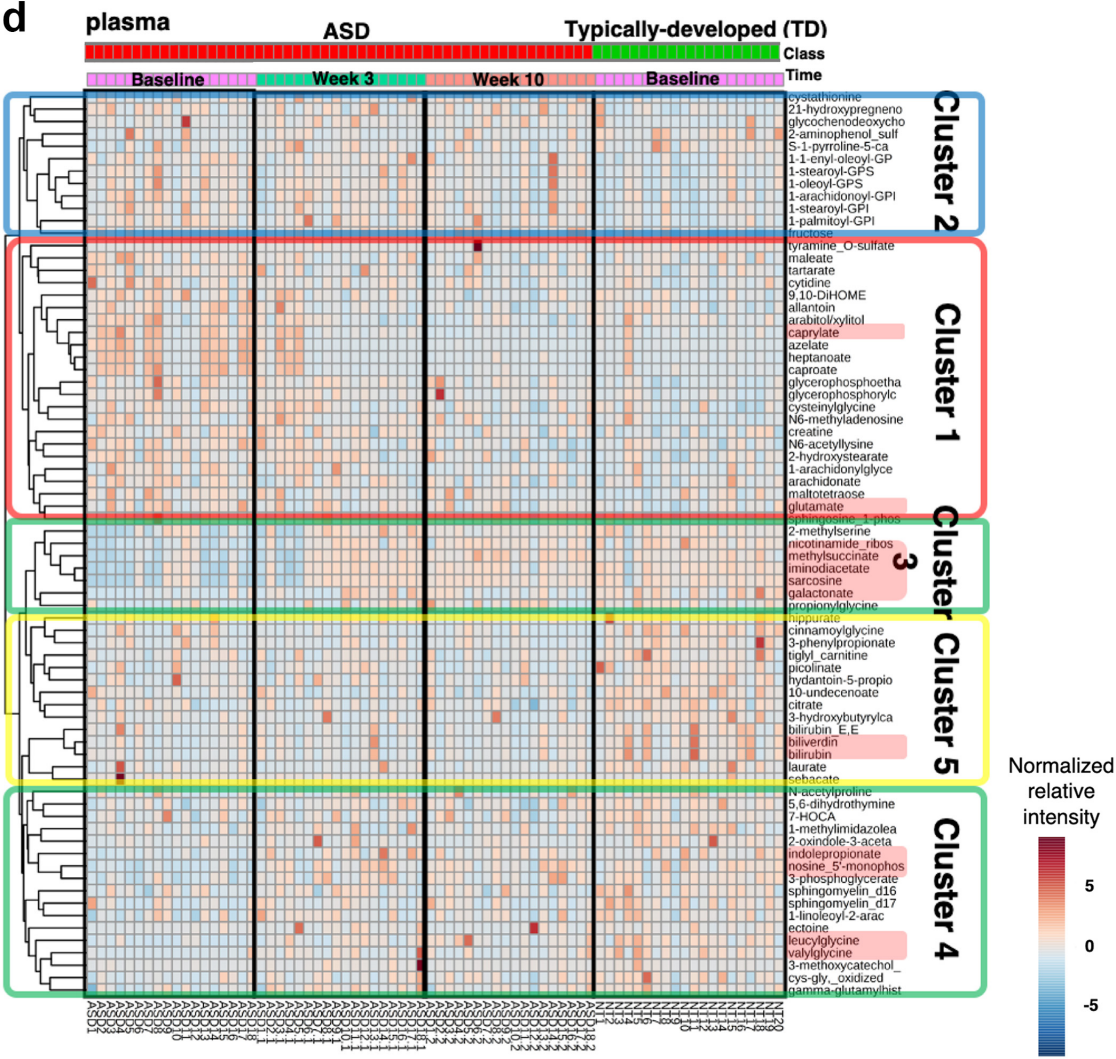

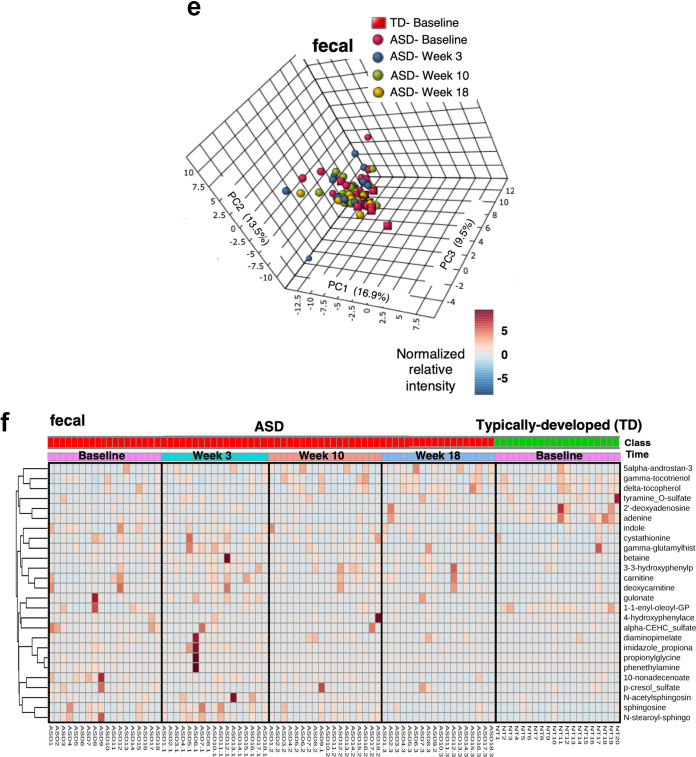
Clinical trial timeline, principal-component analysis (PCA), and heat map profile with dendrogram with plasma and fecal samples at different time points. (a) The timeline consists of 10-week microbiota transfer therapy (MTT) and an 8-week follow-up observation period. (b and d) Seventy-three plasma metabolites whose levels were relatively different at baseline between two groups (unadjusted *P* < 0.05) were included in the PCA and heat map. (c) Distances from the ASD group to TD group were measured by pairwise PERMANOVA. In heat map profiles, the top line indicates diagnosis, with red for ASD and green for TD. The second line indicates time (pink, baseline; green, week 3; orange, week 10; blue, week 18). The colors for each point in the heat maps indicate either higher levels (red) or lower normalized relative intensity (blue). (d) Plasma metabolites in the heat map were clustered when their initial levels at baseline were higher (clusters 1 and 2) or lower (clusters 3, 4, and 5) at baseline and whether they increased/decreased (clusters 1, 3, and 4) or were maintained (clusters 2 and 5) after MTT. Clusters 1, 3, and 4 displayed metabolites whose levels generally became more similar to levels in TD samples after MTT at week 10, whereas metabolites in clusters 2 and 5 did not change significantly after treatment. The plasma metabolites discussed in the text are highlighted (red). (e and f) 26 fecal metabolites whose levels were relatively different at baseline between the two groups (unadjusted *P* < 0.05) were included in the PCA and heat map.

Despite a long list of metabolites that are different in children with ASD, there are few data specific to the subset of children with ASD who have chronic GI symptoms. Also, there is limited information available on how potentially important metabolites are modulated after a therapeutic intervention, especially one involving the human gut microbiome. We performed microbiota transfer therapy (MTT) for children with ASD and observed significant improvement in GI and ASD symptoms (Fig. 1a) (28, 29). Intriguingly, microbial diversity increased significantly during MTT and was even higher 2 years later (29). Higher microbial diversity is an approximate barometer of the health of the gut microbiome (30, 31). We also observed significant increases in levels of *Bifidobacteria*, *Prevotella*, and *Desulfovibrio* after MTT (28).

We previously published a paper on a multivariate analysis of the plasma data from children who participated in our MTT study (32). This study reports comprehensive metabolite measurements from children who participated in our MTT study (28, 29), focusing on a univariate analysis of plasma and fecal metabolite data. Specifically, plasma and fecal samples were assessed at baseline in children with ASD with chronic GI problems (chronic constipation and/or diarrhea) versus TD children without GI issues to determine which metabolites were different in the ASD group before treatment. In addition, similar measurements of plasma and fecal metabolites were made at multiple time points during and at the end of MTT in the ASD group and compared with those from the TD group to record changes in metabolites as a consequence of the microbiome-based treatment.

## RESULTS

### Plasma metabolite profiles in ASD versus TD changed after MTT.

A liquid chromatography-mass spectrometry (LC-MS) assay identified a total of 619 metabolites in our plasma samples (see Data Set S1, Tab 1, in the supplemental material). To evaluate differences in metabolite profiles as a group, we generated a heat map and performed a principal-component analysis (PCA) using the 73 plasma metabolites whose levels were relatively different at baseline between the two groups (unadjusted *P* < 0.05). Figure 1b displays the results of the PCA for each participant in the ASD group at 3 time points and for the TD group. The plot shows that the ASD group cluster at baseline was significantly separated from the TD group cluster at baseline (adjusted *P* < 0.01 by permutational multivariate analysis of variance [PERMANOVA]; 12 of 18 ASD samples in leftmost cluster, whereas the TD group is in the rightmost cluster). After vancomycin treatment (at 3 weeks), the ASD group shifted toward the TD group (only 4 of 18 in leftmost cluster) and further shifted toward the TD group after MTT at week 10 (0 of 18 in leftmost cluster) (Fig. 1b and c). The heat map presented in Fig. 1d shows the metabolites’ normalized relative intensities for the ASD group at three different time points and the normalized relative intensity of the TD group at baseline. As shown in the heat map with the dendrogram in Fig. 1d, the 73 plasma metabolites were clustered into two groups, based on whether they were initially higher (clusters 1 and 2) or lower (clusters 3, 4, and 5) at baseline and whether they increased/decreased (clusters 1, 3, and 4) or were maintained (clusters 2 and 5) after MTT. Cluster 1 (Fig. 1d) included 23 metabolites which displayed higher levels of normalized relative intensity in ASD samples at baseline and became more similar to levels in TD samples after MTT at week 10. Cluster 2 included 12 metabolites that were initially higher in the ASD samples but did not change significantly after treatment. Clusters 3 and 4 (defined as identical but placed separately in Fig. 1d) consisted of 25 metabolites which were lower in the ASD samples at baseline and increased after MTT. Metabolites in cluster 5 were lower at baseline but unchanged after MTT.

10.1128/mSphere.00314-20.3 DATA SET S1 (Tab 1) A total of 619 plasma metabolites identified by LC-MS assay. (Tab 2) A total of 669 fecal metabolites identified by LC-MS assay. (Tab 3) A total of 116 microbial function modules predicted by PICRUSt (phylogenetic investigation of communities by reconstruction of unobserved state) and HUMAnN (the HMP unified metabolic analysis network) analyses. Download Data Set S1, XLSX file, 1.4 MB. Copyright © 2020 Kang et al. 2020 Kang et al. This content is distributed under the terms of the Creative Commons Attribution 4.0 International license.

### Individual metabolite levels in ASD plasma samples were significantly different at baseline.

To identify potentially important metabolites, we performed Mann-Whitney *U* tests for all 619 plasma metabolites and corrected their *P* values for multiple hypotheses by using two separate multiple-hypotheses correction tests (Benjamini-Hochberg and leave-one-out methods). Of 619 metabolites, 9 plasma metabolites were significantly different at baseline between the ASD and TD groups by both correction tests (Table 1) (adjusted *P* < 0.05). Nicotinamide riboside and IMP were the two most significantly different metabolites at baseline based on Benjamini-Hochberg and leave-one-out correction, and both were significantly lower in the ASD group than in the TD group. Amino acid products (sarcosine and methylsuccinate), dipeptides (valylglycine and leucylglycine), galactonate, and iminodiacetate were significantly lower in ASD samples, whereas caprylate (medium-chain fatty acids with 8 carbon atoms; C_8_) was significantly higher in ASD samples (Table 1) (adjusted *P* < 0.05).

**TABLE 1:** Normalized plasma metabolites that were significantly different at baseline after correcting for multiple hypotheses (adjusted *P* < 0.05)

Super pathway	Subpathway	Biochemical	Adjusted *P* value^*a*^		Median	
			BH	Leave one out	ASD	TD
Amino acid	Glycine, serine, and threonine metabolism	Sarcosine	0.04	0.02	−1.70	0.62
	Leucine, isoleucine, and valine metabolism	Methylsuccinate	0.02	0.01	−1.16	0.09
Carbohydrate	Fructose, mannose, and galactose metabolism	Galactonate	0.03	0.01	−0.90	0.46
Cofactors and vitamins	Nicotinate and nicotinamide metabolism	Nicotinamide riboside	0.02	<0.01	−1.03	0.65
Lipid	Medium-chain fatty acid	Caprylate (8:0)	0.05	0.05	1.13	−0.54
		Heptanoate (7:0)	0.05	0.03	1.66	−0.54
Nucleotide	Purine metabolism, (hypo)xanthine/inosine containing	IMP	0.02	<0.01	−1.00	0.36
Peptide	Dipeptide	Leucylglycine	0.04	0.02	−1.01	0.38
		Valylglycine	0.04	0.05	−0.70	0.31
Xenobiotics	Chemical	Iminodiacetate	0.03	0.01	−1.54	0.51

a All *P* values presented here are smaller than 0.05 by two-tailed Mann-Whitney *U* test after correcting for multiple hypotheses. BH, Benjamini and Hochberg (a multiple-hypotheses correction test).

Notably, most metabolites listed above significantly changed after treatment. Sarcosine, methylsuccinate, nicotinamide riboside, IMP, leucylglycine, and iminodiacetate were significantly lower in ASD samples at baseline and increased significantly after vancomycin at week 3 and remained increased after MTT at week 10 compared to baseline (Table 2 and Fig. 1d) (one-tailed Wilcoxon rank test *P* < 0.05, clusters 3 and 4). In contrast, caprylate and heptanoate were significantly higher in ASD samples at baseline and significantly decreased after MTT at week 10 (*P* < 0.05; cluster 1) (Fig. 1d). Only two metabolites (galactonate and valylglycine) in Table 2 did not change significantly. Galactonate was significantly lower in the ASD group than in the TD group at baseline but did not increase after MTT. Valylglycine was significantly lower in the ASD group than in the TD group at baseline, increased at week 3, but returned to its original level at week 10 (Tables 1 and 2). Overall, 8 of 10 abnormal metabolites shifted significantly after MTT so that they became closer to the levels in the TD group, and the other two partially shifted.

**TABLE 2:** Ratios of normalized ASD plasma metabolites to those in TD samples at baseline that were significantly different when comparing baseline to week 3 and week 10

Super pathway	Subpathway	Biochemical	Median ratio (ASD/TD_baseline_)			Adjusted *P* value vs baseline^*a*^	
			Baseline	Week 3	Week 10	Week 3	Week 10
Amino acid	Glycine, serine, and threonine metabolism	Sarcosine	0.15	1.00	0.97	**0.04**	**0.03**
	Leucine, isoleucine, and valine metabolism	Methylsuccinate	0.58	1.07	1.23	**0.03**	**<0.01**
Carbohydrate	Fructose, mannose, and galactose metabolism	Galactonate	0.05	0.68	0.64	0.12	0.25
Cofactors and vitamins	Nicotinate and nicotinamide metabolism	Nicotinamide riboside	0.42	0.89	0.95	**0.03**	**0.03**
Lipid	Medium-chain fatty acid	Caprylate (8:0)	2.91	1.28	0.90	0.10	**0.03**
		Heptanoate (7:0)	6.67	1.15	0.94	0.13	**0.03**
Nucleotide	Purine metabolism, (hypo)xanthine/inosine containing	IMP	0.41	0.89	1.02	**<0.01**	**<0.01**
Peptide	Dipeptide	Leucylglycine	0.24	0.77	0.74	**0.03**	**0.03**
		Valylglycine	0.22	0.76	0.22	0.05	0.09
Xenobiotics	Chemical	Iminodiacetate	0.54	0.98	0.98	**0.03**	**0.04**

a *P* values are written in bold when they are less than 0.05 by one-tailed Wilcoxon signed-rank test after correcting for multiple hypotheses. To obtain the ratios, each metabolite was normalized such that the median value was 1 in the TD group at baseline.

Although not significant after multiple-hypotheses correction, several metabolites that were previously reported as abnormal in either humans or an animal model (8, 26, 33–35) were either relatively higher or lower than in the TD group (see Table S1). We were interested in neurotransmitters (glutamate, GABA, dopamine, and serotonin), tyrosine/tryptophan derivatives (such as *p*-cresol, 4-ethylphenyl sulfate [4-EPS], and indole), and antioxidants (such as glutathione, biliverdin, and bilirubin). The levels of normalized relative intensities were relatively higher for glutamate and lower for indolepropionate, biliverdin, and bilirubin in the ASD group at baseline (two-tailed unadjusted *P* < 0.05). After MTT at week 10, glutamate and indole propionate changed toward that in the TD group (Table S1).

10.1128/mSphere.00314-20.1 TABLE S1 Top 73 plasma metabolites initially different between ASD and typically developing (TD) samples at baseline (unadjusted *P* values < 0.05). Download Table S1, XLSX file, 0.02 MB. Copyright © 2020 Kang et al. 2020 Kang et al. This content is distributed under the terms of the Creative Commons Attribution 4.0 International license.

### Fecal metabolites: tyrosine/tryptophan derivatives were different at baseline.

In fecal samples, we identified 669 metabolites in total (Data Set S1, Tab 2). To evaluate differences in metabolite profiles as a group, we first performed PCA and heat map analysis for the 26 metabolites whose levels were most different at baseline between the two groups (unadjusted *P* < 0.05). As shown in Fig. 1e, PCA results showed that the fecal metabolites of the ASD and TD groups were similar at baseline and remained similar after MTT. Figure 1f shows that there were no significant differences between ASD and TD groups at baseline, and no significant change was observed between baseline and after MTT.

We also investigated individual levels of fecal metabolites, but of 669 metabolites, no metabolite was significantly different after correcting for multiple hypotheses (adjusted *P* > 0.05). Although not statistically significant, three tyrosine derivatives (*p*-cresol sulfate, tyramine *O*-sulfate, and 4-hydroxyphenylacetate) and indole, a serotonin precursor, were relatively different at baseline (unadjusted *P* < 0.05) (see Table S2). *p*-Cresol sulfate, 4-hydroxyphenylacetate, and indole levels were relatively higher in feces from the ASD group at baseline, whereas tyramine *O*-sulfate, a metabolite that has similar chemical structure to those of 4-EPS and *p*-cresol sulfate, was relatively lower in feces from the ASD group. After MTT, *p*-cresol sulfate levels were lowered to levels similar to those of the TD group, and the other metabolites mentioned above did not change toward those in the TD group (Table S2).

10.1128/mSphere.00314-20.2 TABLE S2 Top 26 fecal metabolites initially different between ASD and typically developing (TD) samples at baseline (unadjusted *P* values < 0.05). Download Table S2, XLSX file, 0.01 MB. Copyright © 2020 Kang et al. 2020 Kang et al. This content is distributed under the terms of the Creative Commons Attribution 4.0 International license.

### PICRUSt/HUMAnN analyses predicted microbial functions but not significance.

With 16S rRNA gene amplicon sequencing data (28), PICRUSt (phylogenetic investigation of communities by reconstruction of unobserved state) (36) and HUMAnN (the HMP unified metabolic analysis network) (37) analyses predicted 116 microbial function modules in total throughout the samples (Data Set S1, Tab 3). However, none of the functional modules identified showed statistical significance after correcting for multiple-hypotheses testing.

### Correlation-based networks showed links between metabolites, microbes, and clinical assessments.

**FIG 2:**
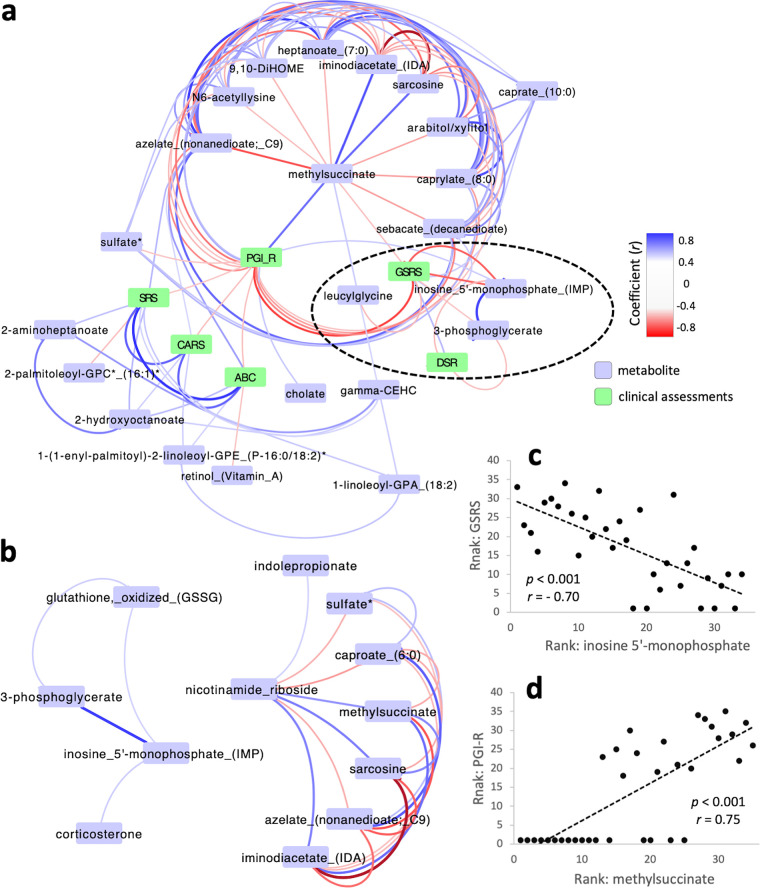
Correlation-based network analysis and correlation tests. Correlation-based network analysis with plasma metabolites associated with clinical assessment scores (highlighted in green) (a) and metabolite pairs that are significantly correlated with nicotinamide riboside and IMP (b). Blue and red lines between metabolites indicate positive and negative correlations, respectively (Spearman correlation coefficient |*r*| > 0.5). Rank-based plots to show strong correlations between GSRS and IMP (c) and PGI-R and methylsuccinate (d). *P* and *r* values are for rank-based Spearman correlation tests.

Figure 2a displays the plasma metabolites that had the most significant correlations (Spearman correlation coefficient |*r*| > 0.5) with clinical assessments of GI and ASD-related symptoms. IMP levels were strongly negatively correlated with GI assessments via the gastrointestinal symptom rating scale (GSRS) and daily stool record (DSR) (a black-dotted circle in Fig. 2a and c); in other words, the increase of IMP levels to normal levels after MTT was strongly associated with improvements in GI symptoms. Methylsuccinate was strongly correlated with parental global impressions-revised (PGI-R) (Spearman correlation, |*r*| = 0.75) (Fig. 2a and d), i.e., the increase of methylsuccinate to normal levels after MTT was strongly associated with improvements in ASD-related symptoms. For plasma metabolites and the three gut bacteria (*Bifidobacterium*, *Prevotella*, and *Desulfovibrio*), we only found one significant correlation between *Bifidobacterium* and 5-(galactosylhydroxy)-l-lysine (*r* = 0.54, *P* < 0.01).

**FIG 3:**
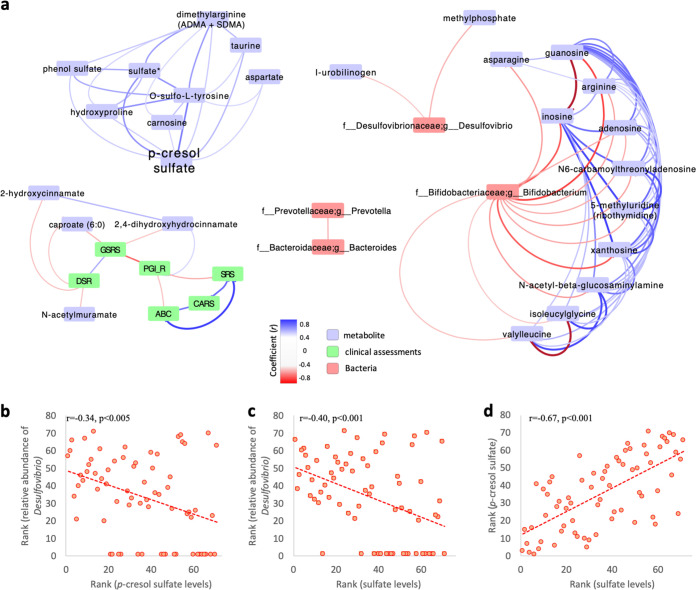
Correlation-based network analysis and correlation tests between fecal metabolites, clinical measurements, and bacterial components in stool. (a) Correlation-based network associated with *p*-cresol sulfate, clinical measurements, and key significant bacteria of *Bifidobacterium*, *Prevotella*, and *Desulfovibrio*. (b to d) Sulfate-reducing *Desulfovibrio*, *p*-cresol sulfate, and sulfate levels were significantly correlated (Spearman correlation, *P* < 0.005).

Figure 3a shows a correlation-based network analysis between fecal metabolites, clinical measurements, and three bacteria (*Bifidobacterium*, *Prevotella*, and *Desulfovibrio*), which were the three gut microbes which significantly changed in response to MTT (28). Correlation-based network analyses shows that *Bifidobacterium* is strongly correlated with many stool metabolites, especially negatively correlated with amino acids/peptides (arginine, asparagine, isoleucylglycine, and valylleucine) and nucleotides (adenosine, xanthosine, and inosine) (Fig. 3a). In contrast, sulfate-reducing *Desulfovibrio* was not strongly correlated with metabolites of interest, but there was a modest significant negative correlation with *p*-cresol sulfate and sulfate (Fig. 3b and c). *p*-Cresol sulfate and sulfate levels were strongly and significantly correlated with each other (Fig. 3d). GI and behavioral symptoms were associated with several metabolites, but those metabolites were not correlated with any of the three bacteria, suggesting that other bacteria (or subsets of those bacteria) are affecting the metabolites associated with GI and ASD symptoms.

## DISCUSSION

**FIG 4:**
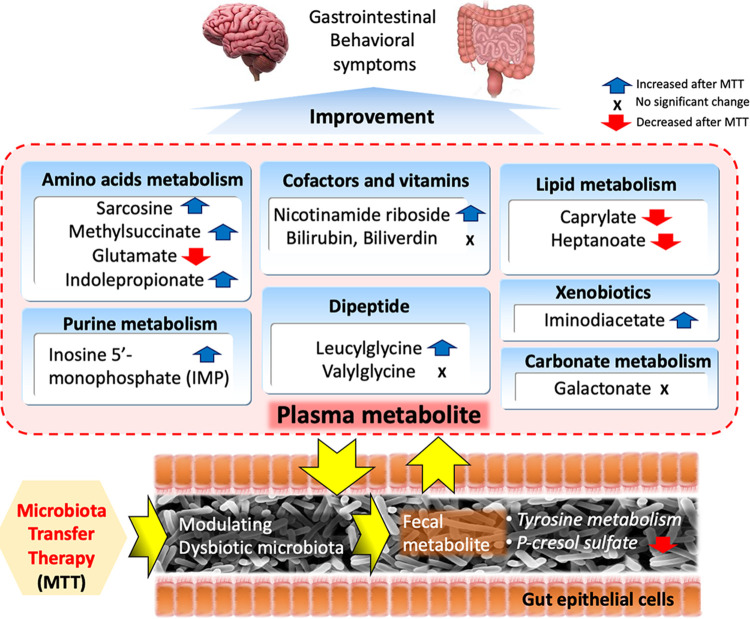
Possible metabolic features that microbiota transfer therapy (MTT) induced for the improvement on gastrointestinal and behavioral symptoms in children with ASD.

Participants in the MTT trial (28, 29) experienced major changes in their gut microbiomes. MTT involved using vancomycin (an antibiotic), a stomach acid suppressant, and a bowel cleanse to reduce the initial microbiome, and adding new microbiota from very healthy donors by fecal microbiota transplant daily for 7 to 8 weeks. This study demonstrates that MTT resulted in major changes in plasma metabolite profiles and modest changes in fecal profiles (summarized in Fig. 4). One reason may be due to the small sample size and greater statistical noise in fecal samples. Another possible hypothesis for the major change in plasma but not fecal metabolites is that perhaps MTT improved epithelial cell integrity and reduced intestinal permeability, resulting in less diffusion of fecal metabolites into the bloodstream and thus a more normal set of plasma metabolites. The other hypothesis is that by modifying the microbiota in the gut, important host interactions that rely on microbial metabolites were also modified, leading to the overproduction or consumption of systemic metabolites.

### Plasma metabolites.

For plasma metabolites, when we consider 75 metabolites whose levels were relatively different at baseline between the two groups (unadjusted *P* < 0.05), PCA found that the ASD group cluster (12 of 18 samples) was significantly separate from the TD group at baseline (adjusted *P* < 0.01 by PERMANOVA), but after vancomycin (week 3) and after 10 weeks of MTT, the ASD group was closer (while not completely similar) to the TD group (Fig. 1b and c). The heat map analysis (Fig. 1d) shows that many individual metabolites that were initially higher (cluster 1, *n* = 23) or lower (clusters 3 and 4, *n* = 25) in ASD shifted toward more normal levels after MTT; however, some metabolites with abnormal levels at baseline did not improve (clusters 2 and 5, *n* = 25). These individual shifts explain changes in the PCA (Fig. 1b). At baseline, 6 children with ASD already had similar plasma metabolite profiles to the one after MTT (Fig. 1b), and those ASD samples (ASD6, ASD9, ASD10, ASD11, ASD12, and ASD16) had lower levels of nicotinamide riboside, methylsuccinate, iminodiacetate, and sarcosine at baseline (in cluster 3) and higher levels of caprylate, azelate, heptanoate, and caproate at baseline (in cluster 1) than the other 12 ASD samples at baseline. At week 3, 8 children with ASD experienced a shift in their plasma metabolites toward those of the TD group, reflecting a potential role of antibiotics and a proton pump inhibitor changing the gut microbiome.

After correcting for multiple hypotheses, we found 10 plasma metabolites that were significantly different in ASD versus controls at baseline (Table 1). Nicotinamide riboside was significantly lower in ASD samples, and their levels significantly increased after MTT (Tables 1 and 2). Nicotinamide riboside is a pyridine nucleoside form of vitamin B_3_ and a precursor for NAD (38, 39), a critical molecule involved in hundreds of redox and other reactions. Typically, tryptophan or aspartate are converted to NAD, but an alternative path involves the synthesis of NAD from nicotinamide riboside (salvage pathways) when the normal pathway is blocked. NAD levels were found to be significantly lower in children with ASD in another study (34) and found to increase significantly after treatment with a vitamin/mineral supplement (22) or by supplementation with NADH or ribose (40). Nicotinamide riboside supplementation prevented neurological damage and improved cognitive and physical function in an Alzheimer’s disease model (41). Intriguingly, nicotinamide riboside in plasma samples is positively correlated with tryptophan derivatives, indolepropionate (or indole-3-propionate), and methylsuccinate (Fig. 2b). Indolepropionate helps maintain mucosal homeostasis and is a neuroprotective antioxidant, which is consistent with our findings of low levels in the ASD group before MTT but normal levels after MTT (42). Methylsuccinate is a competitive inhibitor of succinate dehydrogenase that is involved in the GABA shunt and reactive oxygen stress (ROS) homeostasis (43). Methylsuccinate was strongly positively correlated with PGI-R and negatively correlated with GSRS (*r* = −0.51), consistent with improvements in ASD and GI symptoms after MTT.

IMP and sarcosine levels were significantly lower in the ASD group at baseline and increased after MTT; that increase was strongly correlated with improvements in GI symptoms (GSRS and DSR) (Fig. 2c). We did not find significant correlation between IMP and sarcosine, but these two metabolites together with tyramine *O*-sulfate were the most promising combination to distinguish between TD and ASD with GI symptom groups from a multivariate analysis (32). IMP is involved in NAD-dependent oxidation that converts IMP and NAD^+^ to xanthosine monophosphate and NADH (IMP dehydrogenase). IMP dehydrogenase activity was suggested to be related to irritable bowel disease (44). IMP was significantly and positively correlated with 3-phosphoglycerate (3PG) and oxidized glutathione (Fig. 2b). We did not find a meaningful metabolic relation between these three metabolites, but 3PG is linked to energy generation in mitochondria and folate metabolism (45). Glutathione is an antioxidant which can neutralize ROS and has many important roles in the body, including detoxification and immune function (46). In a previous study (47), reduced glutathione (GSH) levels were lower in plasma samples of children with ASD, and a redox ratio of reduced to oxidized glutathione (GSH:GSSG) was lower than that in typically developing children. In our plasma samples, oxidized glutathione (GSSG) levels were comparable between groups at baseline. Since reduced glutathione was not measured in our analysis, the redox ratio of glutathione was not available to compare with the previous study. Sarcosine, also called *N*-methylglycine, is converted to glycine that has a vital wide-ranging role in human physiology, including in nucleic acid synthesis and central nervous system homeostasis (48). Sarcosine supplementation is beneficial for depression and schizophrenia (49).

Caprylate and heptanoate are medium-chain fatty acids (MCFAs) that were highly correlated with one another (*r* = 0.79). The ASD group had average levels of caprylate and heptanoate at baseline that were 190% and 590% above normal, respectively, and decreased to normal levels after MTT. They were both significantly correlated with the PGI-R (*r* = −0.58 and −0.66), so that their decrease after MTT was correlated with an improvement in ASD symptoms. Galactonate is formed by oxidation of galactose, possibly by galactose dehydrogenase (50). At baseline, the level of galactonate was significantly lower in the ASD group, suggesting impaired production. Another study, however, found that galactonate was higher in children with ASD (51).

Glutamate levels were relatively higher in ASD plasma samples at baseline. This observation is consistent with what we expected, because glutamate is a major excitatory neurotransmitter that has been related to anxiety and excitation, which are behavioral symptoms that children with ASD experience (52, 53). Bilirubin and its oxidized form biliverdin possess antioxidant properties (54, 55), and their relative intensities were relatively lower in plasma samples from the ASD group at baseline (see Table S1 in the supplemental material), reflecting a deficit for preventing oxidative stress in children with ASD (56–58). MTT, however, did not change bilirubin and biliverdin levels toward those in TD group plasma samples (Table S1); therefore, these may be a useful target for other future therapies.

### Fecal metabolites.

Although not significant after controlling for multiple-hypotheses testing, *p*-cresol sulfate levels were relatively higher in the ASD group and decreased toward normal levels after MTT (see Table S2), which is in line with what we expected. Elevated *p*-cresol sulfate induces oxidative stress (59), and tyrosine metabolism along with *p*-cresol has been implicated in the etiology of ASD through human and animal studies (60, 75). A previous study with an autism mouse model claimed *p*-ethylphenyl sulfate (4-EPS) as an important metabolite associated with autism symptoms and pointed out its similar chemical structure to that of *p*-cresol sulfate, a uremic toxin (26). Axial Biotherapeutics has reported elevated concentrations of 4-EPS in children with ASD (61). We previously reported higher *p*-cresol concentrations in ASD fecal samples (8), and in this study, we observed higher but not statistically significant *p*-cresol levels in ASD fecal samples (Table S2). Notably, the decrease in *p*-cresol sulfate after MTT was modestly but significantly associated with the increase in *Desulfovibrio* (Fig. 3b). *Desulfovibrio* has not been listed as a beneficial microbe but rather has been accepted as a detrimental one in the view of the autism phenotype (11, 13, 18, 62); however, we found it significantly increased after MTT at week 10, suggesting it may have some beneficial role (28). Significant correlations between *Desulfovibrio*, *p*-cresol sulfate, and sulfate (Fig. 3b to d) support a potential role for sulfate-reducing microbes such as *Desulfovibrio* in the metabolism of *p*-cresol sulfate (63). Regarding 4-EPS, we only identified 4-EPS in plasma samples, and its levels were marginally lower in ASD samples (two-tailed Mann-Whitney *U* test *P* = 0.09). This observation is not consistent with Hsiao et al.’s data on mice (26), and we did not see any change after MTT. A larger study is needed to clarify if 4-EPS is relevant to human ASD.

Overall, it is intriguing that we observed significant changes in metabolic features in plasma metabolites but not much in fecal metabolites, even though we had found substantial changes in gut microbiota after MTT. This distinct result between plasma and fecal samples reflects some of the challenges of working with complex gut microbiota and their metabolites. This study relied on a small sample size with different GI disorder types (e.g., constipation, diarrhea, or abdominal pain), and a larger cohort may help differentiate subtle but important changes in fecal metabolites that could be masked in this study. Although we employed rigorous nonparametric tests for the network analysis, a larger sample size with more time points would help achieve a more robust correlation network in future studies. At baseline, *p*-cresol sulfate was different in our fecal samples. However, gut microbes produce *p*-cresol not *p*-cresol sulfate, and *p*-cresol produced by gut microbes is converted to *p*-cresol sulfate by host sulfation in the liver. Our observation of *p*-cresol sulfate may reflect its release from host to the gut, like bile acids (64), or by the systemic circulation between gut and liver (65). Further studies with multiple analytical assays are warranted to validate our observation of *p*-cresol sulfate.

### Conclusions.

In summary, MTT drove global changes in plasma metabolite profiles in children with ASD. Vancomycin seems to initiate a shift in the plasma metabolite profiles, but microbiota transfer resulted in further shifts and seemed to stabilize the effect of vancomycin. As a result, plasma metabolite profiles in the ASD group were modulated from being distinctly different from those in the TD group to becoming similar to those in the TD group. Among plasma metabolites that were significantly different in ASD at baseline, medium-chain fatty acids (caprylate and heptanoate) were significantly higher at baseline than in the control and significantly decreased after MTT. Nicotinamide riboside, IMP, iminodiacetate, methylsuccinate, leucylglycine, and sarcosine were significantly lower at baseline and increased after MTT. Other metabolites such as galactonate and valylglycine were abnormal at baseline and did not improve after MTT, and so those are potential targets for other future therapies. Overall, these data provide some insight into why microbiota transplant therapy appears to be able to significantly reduce GI and ASD symptoms. It also suggests a biochemical basis for some ASD symptoms.

In contrast, for fecal metabolites, only a few metabolites were different at baseline, and none were significantly different after correction for multiple-hypotheses testing. At baseline, *p*-cresol sulfate, 4-hydroxyphenylacetate, tyramine *O*-sulfate, and indole were relatively different between groups (unadjusted *P* < 0.05), and only *p*-cresol sulfate significantly changed toward that in the TD group after MTT. There were significant correlations between *p*-cresol sulfate, sulfate, and *Desulfovibrio*, suggesting a potential role of *Desulfovibrio* in the metabolism of *p*-cresol sulfate and possible autism etiology. Further studies of fecal and blood metabolites in ASD, before and after MTT, are warranted, as well as clinical trials of other therapies, to address the metabolic changes which MTT was not able to correct.

## MATERIALS AND METHODS

### Ethics approval and consent to participate.

The protocol for the original treatment study was approved by the Institutional Review Board of Arizona State University (ASU IRB protocol 00001053) and the U.S. FDA (investigational new drug number 15886), as described by Kang et al. (28). All plasma and fecal samples were collected from the 20 typically developing children and 18 children with ASD who participated in the original phase 1 trial study in accordance with the relevant guidelines and regulations, and no further inclusion or exclusion criteria were employed in addition to what we applied in the original trial (28). The consent process was initiated by communicating with past participants and their parents via email, and the study was explained in detail by phone and/or email. For the families who met the study criteria, we provided families with a copy of a child assent form and a parent permission form. After the informed consent process, families with children with ASD and typically developing children agreed to participate and signed written parent permission and assent forms. We deidentified all samples and documents for the entire study and maintained participants’ confidentiality for the entire analyses. The trial was registered at ClinicalTrials.gov (NCT02504554) on 30 March 2015.

### Study design and MTT.

The study protocol was approved by the U.S. Food and Drug Administration (FDA) (investigational new drug number 15886) and the Institutional Review Board of Arizona State University (ASU IRB protocols 00001053 and 00004890). The study population and MTT protocol are described in a previous study (28). In summary, 18 children with ASD and 20 typically developing children participated in the study. All 18 children with ASD were 7 to 16 years old and completed the 18 weeks of the open-label trial, consisting of antibiotic (vancomycin) treatment for 2 weeks, a bowel cleanse for 1 day (MoviPrep), a proton pump inhibitor (Prilosec) throughout the trial, and standardized human gut microbiota (66) of a high dose for 1 or 2 days followed by a maintenance dose for 7 to 8 weeks. We monitored participants during the 10 weeks of treatment as well as 8 weeks after MTT stopped. There were no major adverse effects. We observed substantial improvement in their GI and ASD symptoms and an increase in microbiome diversity. Further information on the clinical and microbial observations are available in the study by Kang et al. (28).

### Sample collection for metabolomic analysis.

For metabolomic analysis, plasma samples were collected at baseline, week 3 (after the vancomycin and bowel cleanse, and just before the first dose of microbiota), and week 10 (the end of MTT treatment). Phlebotomists who had experience with children drew blood samples in the morning from fasting participants, centrifuged them to collect plasma samples, and froze plasma samples immediately in dry ice. Frozen samples were delivered to the laboratory and stored in a −80°C freezer until all samples were collected. Plasma samples were collected from all 18 children with ASD at three time points except for one missing sample at week 3. Plasma samples were collected from all 20 TD participants at baseline only, since they did not receive MTT. To be consistent with microbial analysis (28), we used the same fecal samples as those by Kang et al. (28) collected at baseline, week 3 to 4 (approximately 6 days after the initial high dose of microbiota), week 10, and week 18 (8 weeks after the end of MTT) for children with ASD and baseline for typically developing children. Fecal samples collected by participants at home were immediately frozen and then picked up by a student driver and delivered to the laboratory on dry ice to be stored in a −80°C freezer. Once the clinical trial concluded, the frozen samples were aliquoted and sent overnight with dry ice to Metabolon (Durham, NC, USA) for metabolomic analysis. To avoid any bias in metabolomics measurement, we randomized and blinded all autism and control samples in a random order before shipping out to Metabolon.

### Metabolomic analysis and sample normalization.

At Metabolon, a platform with ultrahigh performance liquid chromatography-tandem mass spectroscopy (UHPLC-MS/MS) instruments was employed. Metabolomic measurements consisted of sample extraction/preparation, quality assurance/quality control, and UHPLC-MS/MS at Metabolon, Inc., as described in detail elsewhere (67–70). Briefly, the samples were processed by mixing with methanol, vigorous strokes, and centrifugation, which allowed protein precipitation and various metabolite recovery in the supernatant (68, 70). The extracts were aliquoted, dried, and reconstituted in different solvents depending on the platform for either polar or nonpolar metabolite analyses (hydrophilic interaction liquid chromatography [HILIC]/UPLC-MS/MS or reverse-phase [RP]/UPLC-MS/MS, respectively) (69). The organic solvent was then removed by evaporation using a TurboVap (Zymark, Hopkinton, MA). The sample extracts were analyzed by the methods utilizing a Waters ACQUITY UPLC (Milford, MA) and a Thermo Scientific Q-Exactive high-resolution/accurate mass spectrometer interfaced with a heated electrospray ionization (HESI-II) source and Orbitrap mass analyzer (Thermo Scientific, Waltham, MA). Metabolon generated deliverables, including chemical annotation, the retention time index (RI), mass, and raw area counts after metabolite quantitation. Metabolite quantitation was performed by peak area integration using the area under the curve, and the intensity measurements yielded relative intensity of each metabolite. To correct the systematic variation between measurements, data normalization was conducted at Metabolon Inc. by registering the medians to equal 1 and normalizing each data point proportionately (called the block correction). Missing values were imputed with the lowest value of each compound measurement divided by the square root of 2. With the metabolomics data sets after imputation, we normalized the relative intensity into a Z-score, which is a dimensionless quantity, to obtain a mean value of 0 and standard deviation of 1 for each individual metabolite. The Z-score indicates how many standard deviations the observation is above or below the mean and is useful to compare observations coming from different distributions of individual metabolites. Throughout the manuscript, we call these values normalized relative intensity and report all data comparing the two groups as normalized relative intensity. To calculate the ratio of the ASD group at different sampling points to the TD group at baseline (ASD/TD_baseline_), each metabolite value was normalized such that the median value was set to a value of 1.0 in the TD cohort. In detail, we first obtained a median value of the TD group for each metabolite and divided all individual values of the ASD group by the TD median value. Then, we calculated each median value of the ASD group at each time point (baseline, week 3, and week 10). With those median values, we obtained the ratio between the ASD and TD groups by dividing each ASD median value by the TD median value (ASD_baseline_/TD_baseline_, ASD_week3_/TD_baseline_, and ASD_week10_/TD_baseline_).

### Bioinformatics and statistical analysis.

To elucidate the metabolite profiles as a group, heat map analysis and principal-component analysis (PCA) were performed using the MetaboAnalyst 4.0 (71). To determine significant differences between groups, we first measured distances by the weighted Bray-Curtis matric and then performed a permutation-based nonparametric method, PERMANOVA (permutational multivariate analysis of variance), using QIIME2’s command “beta-group-significance” (72). To control multiple comparisons, we conducted 999 permutations and adjusted the *P* values by Benjamini-Hochberg false-discovery rate correction. For heat map analysis, the dendrogram was generated by using a hierarchical clustering method with complete linkage and “Euclidean” distance to measure the dissimilarity. To investigate how metabolites were potentially interrelated with the improvement of GI and behavioral symptoms, we performed a correlation-based network analysis with ASD samples at different time points by calculating Spearman correlation tests for ASD plasma metabolites and clinical measurements. We only considered correlations with adjusted *P* values of less than 0.1 after correcting for multiple hypotheses in the correlation-based network. We used Cytoscape to visualize the correlation-based network (73).

Using 16S rRNA gene amplicon sequencing data from the original study (28), we predicted microbial functions using PICRUSt v.1.1.3 (36), HUMAnN v0.99 (37), and linear discriminant analysis with effect size (LEfSe) (74) analyses available in the online Galaxy version of the Huttenhower Lab (v1.0.0). Through PICRUSt, we first obtained predicted metagenome files containing gene counts, and then we employed the PICRUSt output to HUMAnN to obtain gene and pathway summaries based on Kyoto Encyclopedia of Genes and Genomes (KEGG) modules. To investigate which bacterial functional module is associate with ASD phenotype, we performed a linear discriminant analysis with effect size (LEfSe), where the nonparametric factorial Kruskal-Wallis (KW) sum-rank test was used to detect significantly different genomic features with respect to the class of interest, ASD, and then linear discriminant analysis (LDA) was used to estimate the effect size of each individually abundant feature.

We assumed the metabolomic and 16S rRNA gene sequencing data were nonnormally distributed because of a relatively small sample size and employed nonparametric statistical tests with Mann-Whitney *U* test, Wilcoxon signed-rank test, Spearman correlation test, and KW sum-rank test. For metabolites and genomic features, we adjusted *P* values by correcting for multiple hypotheses with Benjamini-Hochberg and leave-one-out methods, and adjusted *P* values smaller than 0.05 were considered statistically significant. For metabolites that were previously reported and hypothesized as important, when unadjusted *P* values were lower than 0.05 but adjusted *P* values were higher than 0.05, we considered them as relatively higher or lower to indicate their relative difference between ASD and TD groups. For paired data (before and after MTT treatment in the same participants), we used the Wilcoxon signed-rank test and one-tailed test, since we hypothesized the direction of either increase or decrease of normalized relative intensity based on the measurements at baseline.

### Data availability.

The 16S rRNA gene sequence data sets are available in the open-source sequence data repository NCBI SRA under BioProject identification (ID) PRJNA529598 and “Qiita” with the study ID number 10532 (https://qiita.ucsd.edu). The clinical data are available in the study by Kang et al. (28). Metabolomic and PICRUSt data sets are included in tables in the supplemental material.
